# Phonological and Semantic Specialization in 9- to 10-Year-Old Children During Auditory Word Processing

**DOI:** 10.1162/nol_a_00099

**Published:** 2023-04-11

**Authors:** Jin Wang, Brianna L. Yamasaki, James R. Booth

**Affiliations:** Department of Psychology and Human Development, Vanderbilt University, Nashville, TN; Harvard Graduate School of Education, Harvard University, Cambridge, MA; Department of Psychology, Emory University, Atlanta, GA

**Keywords:** phonology, semantics, language, specialization, development, children

## Abstract

One of the core features of brain maturation is functional specialization. Previous research has found that 7- to 8-year-old children start to specialize in both the temporal and frontal lobes. However, as children continue to develop their phonological and semantic skills rapidly until approximately 10 years old, it remained unclear whether any changes in specialization later in childhood would be detected. Thus, the goal of the current study was to examine phonological and semantic specialization in 9- to 10-year-old children during auditory word processing. Sixty-one children were included in the analysis. They were asked to perform a sound judgment task and a meaning judgment task, each with both hard and easy conditions to examine parametric effects. Consistent with previous results from 7- to 8-year-old children, direct task comparisons revealed language specialization in both the temporal and frontal lobes in 9- to 10-year-old children. Specifically, the left dorsal inferior frontal gyrus showed greater activation for the sound than the meaning task whereas the left middle temporal gyrus showed greater activation for the meaning than the sound task. Interestingly, in contrast to the previously reported finding that 7- to 8-year-old children primarily engage a general control region during the harder condition for both tasks, we showed that 9- to 10-year-old children recruited language-specific regions to process the more difficult task conditions. Specifically, the left superior temporal gyrus showed greater activation for the phonological parametric manipulation whereas the left ventral inferior frontal gyrus showed greater activation for the semantic parametric manipulation.

## INTRODUCTION

Effective language comprehension and production skills are crucial for success in daily life. According to the interactive specialization theory (Johnson, 2011), developing complex cognitive skills, such as those associated with language use, involves a prolonged process of neural specialization. That is, it is hypothesized that children move through a process during which cortical regions become more functionally tuned, or more responsive to their preferred task or contexts than other tasks or contexts, with a region’s “preferred task” referring to a task (or tasks) that requires a process that a cortical region is selectively sensitive to in the mature brain. While there has only been limited support for this theory within the domain of language, there has been evidence for this process of neural specialization within other cognitive domains such as face processing (e.g., Aylward et al., 2005; Gathers et al., 2004), social cognition (e.g., Carter & Pelphrey, 2006), reading (e.g., Schlaggar & McCandliss, 2007), and cognitive control (e.g., Durston et al., 2006). Furthermore, it has been demonstrated that children with developmental disorders affecting these cognitive processes tend to present with atypical patterns of brain specialization (Johnson, 2011). Understanding the development of language-related neural specialization in typically developing children will not only add to this growing body of research providing empirical support for the interactive specialization theory but will also inform our understanding of the maturational trajectory of the neural systems that support language and will provide a foundation on which to better understand what may be different in children with developmental language disorders.

Successful language use involves the coordination of many different cognitive systems, two of which are the phonological, or sound processing, and semantic, or meaning processing, systems. Decades of theoretical, and supporting empirical work, has identified a unique network of regions that support each of these component skills. More specifically, the left superior temporal gyrus (STG), supramarginal gyrus (SMG), inferior parietal lobule (IPL), and posterior dorsal inferior frontal gyrus (dIFG) have been shown to support phonological processing. Whereas, the left middle temporal gyrus (MTG), angular gyrus (AG), anterior fusiform gyrus (FG), and anterior ventral inferior frontal gyrus (vIFG) have been associated with semantic processing (e.g., Binder et al., 2009; Friederici & Gierhan, 2013; Hickok & Poeppel, 2007). Evidence supporting phonological and semantic specialization within these regions has primarily come from work with adults using direct task comparisons, a statistical approach which cancels out irrelevant processes that are shared between two similar tasks (i.e., phonological and semantic) and thus identifies regions sensitive purely to each individual task (i.e., phonological or semantic processing; see Hodgson et al., 2021, for a recent meta-analysis). Although neurocognitive theories of language development have suggested that children exhibit phonological and semantic sensitivity in the brain as early as the first two years of life (see review in Skeide & Friederici, 2016), the evidence for phonological and semantic specialization in children using the direct task comparison approach is scarce and inconsistent.

Of the few previous studies which have examined phonological and semantic specialization using direct task comparisons in developing children, findings are mixed. Mathur et al. (2020) studied 5- to 7-year-old children but did not find differences in brain activation between a visual rhyming and a visual meaning task. Landi et al. (2010) recruited children who were 9 to 19 years old and asked them to perform a cross-modal (auditory and visual) categorical meaning judgment task and a visual rhyme judgment task. They found evidence of a single dissociation, that is, children engaged the left STG and AG more in the meaning task than the rhyming task but there were no regions that showed greater engagement during the rhyming over the meaning task. Similarly, Liu et al. (2012) recruited children aged 11 to 13 years old and compared their brain activation during a visual rhyming and a visual meaning association task. They too found evidence only for semantic-specific regions, in particular, that the left vIFG and MTG showed greater activation for the meaning than the rhyming task, but there was no evidence for phonology-specific regions.

One critical characteristic of the previously described studies is that they all used visual word stimuli, which may have introduced confounds related to the visual decoding process. Using an alternative, auditory stimuli approach, Weiss et al. (2018) and Wang, Yamasaki, et al. (2021) aimed to investigate language-related neural specialization without the potential confound associated with visual stimuli. Both studies showed support for specialization via a double dissociation. Weiss et al. (2018) found that 5- to 6-year-old children showed greater activation in the left STG during an auditory sound judgment compared to an auditory meaning judgment task and greater activation in the left MTG during an auditory meaning judgment task compared to an auditory sound judgment task. In a slightly older sample, Wang, Yamasaki, et al. (2021) found that 7- to 8-year-old children showed greater activation in the left dIFG during the sound versus meaning judgment task and greater activation in both the left vIFG and MTG during the meaning versus sound judgment task. The progression of language specialization from the temporal lobe to the frontal lobe, as suggested by the findings of Weiss et al. (2018) and Wang, Yamasaki, et al. (2021), is consistent with the neurocognitive theory of language development proposed by Skeide and Friederici (2016), which argues that language processes in the temporal lobe develop earlier than those in the frontal lobe.

Taken together, the limited previous research on phonological and semantic specialization in children is mixed, ranging from no to strong support for language-related specialization. These mixed findings may be driven by methodological differences (e.g., visual vs. auditory tasks) or different age groups selected by different studies. Regardless, it is clear that more work is needed to better understand whether or not, in line with the interactive specialization theory, children show evidence for early neural specialization within the language network. As is reviewed above, previous double-dissociation-based evidence for phonological and semantic specialization has only been found in younger children, aged 5 to 8 years old, and only when auditory tasks were used (i.e., Wang, Yamasaki, et al., 2021; Weiss et al., 2018). No double-dissociation-based evidence has been observed in previous studies examining older children using visual stimuli (i.e., Landi et al., 2010; Liu et al., 2012). Thus, studying a slightly older cohort using auditory tasks may help clarify if older children show both phonological and semantic specialization.

The current study focused on the development of phonological awareness and semantic association skills, both of which require metalinguistic processing in addition to phonological and semantic representations. These two language skills have been shown to be closely associated with children’s reading skills (e.g., Melby-Lervåg et al., 2012; Swart et al., 2017). According to the developmental theory by Anthony and Francis (2005), phonological awareness progresses from large grain sizes, such as syllabic and rhyme awareness, to small grain sizes, such as phonemic awareness. The shift to small grain phonemic awareness usually occurs at 5 to 7 years old, after children learn to read, and becomes stable around 9 to 10 years old (Wagner et al., 1997). Different from phonological processing, which has been shown to be related to children’s status of dyslexia or risk of reading disability (e.g., Gu & Bi, 2020; Noordenbos & Serniclaes, 2015; Snowling & Melby-Lervåg, 2016), phonological awareness places more demand on metalinguistic processing, which is more strongly related to reading skills, especially for older children or skilled readers (e.g., Wang et al., 2020; Wang, Pines, et al., 2021). As for the development of semantic associations, the spreading activation theory by Collins and Loftus (1975) suggests that words with higher frequency of co-occurrence establish stronger links between concept nodes in semantic retrieval, and thus should be earlier to develop. Consistent with this, Unger and Fisher (2021) argue that exposure to co-occurrence regularities is the driving force for semantic knowledge development. Children’s vocabulary growth rate has been shown to slow down at approximately 10 years old (Rice & Hoffman, 2015). Thus, as compared to 5- to 8-year-old children, who are experiencing shifts from child to adult-like phonological and semantic processing, 9- to 10-year-old children are near adult-like, providing an ideal age range to examine the potential emergence of adult-like specialization within the language network.

Using the same experimental design and analytical approach as in Weiss et al. (2018) and Wang, Yamasaki, et al. (2021), the goal of the current study was to examine phonological and semantic specialization in 9- to 10-year-old children during auditory word processing. Based on cognitive models of language processing (e.g., Binder et al., 2009; Friederici & Gierhan, 2013; Hickok & Poeppel, 2007; Skeide & Friederici, 2016) and previous findings of neural specialization in children (e.g., Landi et al., 2010; Liu et al., 2012; Mathur et al., 2020; Wang, Yamasaki, et al., 2021; Weiss et al., 2018), we expected that 9- to 10-year-old children would show phonological and semantic specialization in both the frontal and temporal lobe using direct task comparisons. Specifically, we hypothesized that the auditory meaning judgment task would elicit greater activation than the auditory sound judgment task in semantically related regions such as the left IFG, MTG, AG, and/or FG, whereas the auditory sound judgment task would elicit greater activation than the auditory meaning judgment task in phonologically related regions such as the left IFG, STG, and/or SMG. Within the left IFG, it was predicted that the peak of activation for the sound greater than meaning task contrast would be more dorsal than the peak of activation for the meaning greater than sound task contrast.

In addition to direct task comparisons, a hard and an easy condition within each task (onset vs. rhyme and low vs. high association within the sound judgment and meaning judgment tasks, respectively) was designed. The onset condition is predicted to be more difficult than the rhyme condition because the former requires the recognition of smaller grain sizes at the phoneme level which are acoustically less salient and develop later (Anthony & Francis, 2005). The low association condition is predicted to be more difficult than the high association condition because the former has a lower frequency of co-occurrence (Unger & Fisher, 2021). We contrasted the hard and easy conditions to examine whether the specialized regions for each task were also sensitive to within-task differences in difficulty levels. A previous meta-analysis on adults (Hodgson et al., 2021) showed that during more difficult language tasks both a domain-general region (i.e., the opercular part of the left IFG), which is active regardless of task, and language-specific regions (i.e., the orbitalis and triangular part of the left IFG), which are only responsive to certain language tasks, were engaged. Similarly, Wang, Yamasaki, et al. (2021) showed in 7- to 8-year-old children that the opercular part of the left IFG was more active for the hard than easy condition regardless of task, whereas the left STG was uniquely activated more for the onset than rhyme condition in the sound judgment task. Therefore, we hypothesized that 9- to 10-year-old children would also show both domain-general and language-specific regions engaged during the parametric manipulations. Specifically, consistent with the predictions for the direct task comparisons, we expected that regions such as the left IFG, STG, and/or SMG would show stronger activation for the onset than the rhyme condition during the auditory sound judgment task, whereas regions such as the left IFG, MTG, AG, and/or FG would show stronger activation for the low than the high association condition during the auditory meaning judgment task. It was predicted that the peak of activation in the left IFG for the low greater than high association contrast would be more ventral than the peak in the left IFG for the onset greater than rhyme contrast. In addition, we hypothesized that a domain general control region (i.e., the opercular part of the left IFG) would show stronger activation for the onset and low association conditions during the auditory sound and meaning judgment tasks, respectively. All of the above hypotheses and the analytic approach outlined below were preregistered at https://osf.io/5p3es/.

## MATERIALS AND METHOD

### Participants

Data for this study were pulled from a shared data set on OpenNeuro.org (see data descriptor by Wang et al., 2022; https://openneuro.org/datasets/ds003604). The specific subjects and runs used in the current study as well as the code used to analyze the data were shared on GitHub (https://github.com/wangjinvandy/PhonSem_Specialization_9_10). The Institutional Review Board of the University of Texas, Austin, approved all the experimental procedures. Consent was collected from participants’ parents or guardians and assent was collected from children before participation in our study.

Parents or guardians were asked to complete an exclusionary survey and a developmental history questionnaire. All participants enrolled had normal hearing and normal/corrected-to-normal vision, and had no learning, neurological, or psychiatric disorders. Children were asked to complete several screening tests, which included five handedness questions, in which the child had to pretend to write, erase, pick-up, open, and throw something, and the Diagnostic Evaluation of Language Variation (DELV) Part 1 Language Variation Status (Seymour et al., 2003). Children also completed standardized tests to assess their language skills and nonverbal IQ. General language skill was measured using the Clinical Evaluation of Language Fundamentals—Fifth Edition (CELF-5; Wiig et al., 2013). Phonological skill was assessed using the Comprehensive Test of Phonological Processing (CTOPP-2; Wagner et al., 2013). Nonverbal IQ was measured using the Kaufman Brief Intelligence Test—Second Edition (KBIT-2; Kaufman & Kaufman, 2004).

One hundred and one 9- to 10-year-old children participated in the sound and meaning judgment functional magnetic resonance imaging (fMRI) tasks, and those who met the following seven criteria were included in the analysis in the current study: (1) complete data obtained for both runs of the sound and meaning tasks (16 excluded); (2) right-handed, defined as completing at least three out of the five handedness tasks with their right hand (0 excluded); (3) a mainstream American English speaker as categorized by the Part I Language Variation Status subtest on the DELV (0 excluded; this criterion was used because dialects may affect children’s perception of phonology in spoken words); (4) a standardized IQ score of 80 or higher on the KBIT-2 (6 excluded); (5) typical language abilities, as indexed by a standardized Core Language Scale score of 80 or higher on the CELF-5 (0 excluded); (6) no excessive movement during the fMRI tasks (5 excluded, see Data Analysis for criteria); and (7) good fMRI task accuracy (13 excluded, see Experimental Procedure for criteria). In addition, the language test (i.e., CELF-5), which is more interactive, was always the first standardized test administered. In the end, 61 children (37 females, 24 males, mean age = 9.20, *SD* = 0.19, range = 8.96 to 9.87 yr old) were included in the final sample for this study. (Note, we re-analyzed the data by adding 4 participants who had low IQ scores. We added 4 participants, rather than 6, because one participant was left-handed, and one had low in-scanner accuracy. We found that all findings remained the same except that the cluster in the left STG (*k* = 32) was no longer significant for the contrast of Onset > Rhyme after family-wise error, or FWE, correction.) Among the participants, 33 children were included in a previous study examining phonological and semantic specialization in 7- to 8-year-old children (Wang, Yamasaki, et al., 2021) and nine children were included in a previous study on phonological and semantic specialization in 5- to 6-year-old children (Weiss et al., 2018).

### Experimental Procedure

#### The sound judgment task

The sound judgment task taps into children’s phonological processing skill for spoken words. In this task, participants heard a one-syllable word pair presented sequentially through earphones. Children were asked to judge whether the word pair shares any of the same sounds. Real words were used in the sound judgment task instead of the more traditional use of pseudowords, which might induce semantic processing in addition to phonological processing. However, we designed the task in this way because the aim was to use a direct task comparison approach to isolate the regions that were specifically recruited for phonological or semantic processing, so the type of word had to be kept constant between the sound and meaning judgment tasks. The sound judgment task included three different experimental conditions: rhyme, onset, and unrelated (see Table 1 for examples, and see a full list of stimuli in the Supporting Information). Children were expected to press the “yes” button for both the onset and rhyme conditions and the “no” button for the unrelated condition. In addition to the three experimental conditions, the task also included a perceptual control condition in which participants heard two sequentially presented frequency-modulated sounds (i.e., “shh-shh”) and were only asked to press the “yes” button. Participants completed two runs of the task with 12 trials per condition per run for a total of 24 trials for each of the four conditions. The task included a total of 96 trials divided into two separate 48-trial runs. Each auditory word had a duration ranging from 439 to 706 ms. The second word was presented approximately 1,000 ms after the onset of the first word. Overall, within each trial, the stimuli duration (i.e., the two words with a brief pause in between) ranged from 1,490 to 1,865 ms and was followed by a jittered response interval ranging from 1,500 to 2,736 ms. A blue circle appeared simultaneously with the auditory presentation of the stimuli to help maintain attention on the task. The blue circle changed to yellow, to provide a 1,000 ms warning for participants to respond if they had not already done so, before moving on to the next trial. The total trial duration ranged from 3,000 to 4,530 ms. Each run lasted approximately 3 min.

**Table 1.** Experimental conditions in the sound and meaning judgment tasks

Task	Condition	Response	Brief explanation	Example
Sound task	Onset	Yes	Two words share the first sound	Coat–Cup
	Rhyme	Yes	Two words share the final sound	Wide–Ride
	Unrelated	No	Two words do not share sounds	Zip–Cone
	Perceptual	Yes	Frequency modulated noise	Shh–Shh
Meaning task	Low	Yes	Two words are weakly associated in meaning	Dish–Plate
	High	Yes	Two words are strongly associated in meaning	Dog–Cat
	Unrelated	No	Two words are not related in meaning	Map–Hut
	Perceptual	Yes	Frequency modulated noise	Shh–Shh

*Note*. In the sound judgment task, children were asked: “Do the two words share any of the same sounds?” In the meaning judgment task, children were asked: “Do the two words go together?”

The auditory word conditions were designed according to the following standards. For the onset condition, the word pairs shared the same initial phoneme (corresponding to one letter at the beginning of their written form). For the rhyme condition, the word pairs shared the same final vowel and phoneme/cluster (corresponding to two to three letters at the end of their written form). For the unrelated condition, there were no shared phonemes at any locations in a word pair although one letter of the written form may be shared on some occasions (e.g., land–face). All words were monosyllabic, and all word pairs had no semantic association based on the University of South Florida Free Association Norms (Nelson et al., 2004). There were no significant differences between conditions in word length, number of phonemes, written word frequency, orthographic neighbors, phonological neighbors, semantic neighbors, or number of morphemes (Rhyme vs. Onset: *p*s > 0.123; Rhyme or Onset vs. Unrelated: *p*s > 0.123; linguistic characteristics were obtained from the English Lexicon Project by Balota et al., 2007). There were also no significant differences between conditions in phoneme probabilities obtained from a phonotactic probability calculator (Rhyme vs. Onset: *p*s > 0.302; Rhyme or Onset vs. Unrelated: *p*s > 0.203; Vitevitch & Luce, 2004).

#### The meaning judgment task

The meaning judgment task examines children’s semantic processing skill for spoken words. In this task, participants heard a one- or two-syllable word pair presented sequentially through earphones. They were asked to determine whether the word pair goes together semantically. The task included three different experimental conditions: low association, high association, and unrelated (see Table 1 for examples, and see a full list of stimuli in the Supporting Information). Children were expected to press the “yes” button for both the low and high association conditions and the “no” button for the unrelated condition. In addition to the three experimental conditions, the task included a perceptual control condition in which participants heard two sequentially presented frequency-modulated sounds (i.e., “shh-shh”) and were only asked to press the “yes” button. Participants completed two runs of the task with 12 trials per condition per run for a total of 24 trials for each of the four conditions. The task included a total of 96 trials divided into two separate 48-trial runs. Each auditory word had a duration ranging from 500 to 700 ms. The second word was presented approximately 1,000 ms after the onset of the first word. Overall, within each trial, the stimuli duration (i.e., the two words with a brief pause in between) ranged from 1,500 to 1,865 ms and was followed by a jittered response interval ranging from 1,800 to 2,701 ms. A blue circle appeared simultaneously with the auditory presentation of the stimuli to help maintain attention on the task. The blue circle changed to yellow, to provide a 1,000 ms warning for participants to respond if they had not already done so, before moving on to the next trial. The total trial duration ranged from 3,300 to 4,565 ms. Each run lasted approximately 3 min.

The auditory word conditions were designed according to the following standards. Words, with relatively high frequencies, were selected from the database of the University of South Florida Free Association Norms (Nelson et al., 2004). The low and high association semantic relationships were determined using the forward cue-to-target strength (FSG) values reported from the norm. The low association condition was defined as word pairs having a weak semantic association with FSG values between 0.14 and 0.39 (mean = 0.27, *SD* = 0.07). The high association condition was defined as word pairs having a strong semantic association with FSG values between 0.40 and 0.85 (mean = 0.64, *SD* = 0.13). The unrelated condition was defined as word pairs that had no FSG values. The FSG values reflect the proportion of subjects in the group who produce a particular target in the presence of the cue word. The norming is not based on children, and therefore, is a limitation of the design in this study. However, as can be seen below, the behavioral data from this study showed that children responded more poorly to the low association word pairs, which suggests that the intended manipulation of semantic association strength was successful in our sample of young children (see statistics in Results). There were no significant differences in association strength between the two runs of the meaning judgment task (*p*s > 0.425). There were also no significant differences between conditions in word length, number of phonemes, number of syllables, written word frequency, orthographic neighbors, phonological neighbors, semantic neighbors, or number of morphemes (High vs. Low: *p*s > 0.167; High or Low vs. Unrelated: *p*s > 0.068; linguistic characteristics were obtained from the English Lexicon Project by Balota et al., 2007).

Participants who scored within an acceptable accuracy range and demonstrated no response bias on the fMRI tasks were included in the final analysis. Specifically, to be included, children had to score greater than or equal to 50% on the perceptual and rhyme/high conditions (to ensure that children were engaged in and capable of performing the tasks), and children had to have an accuracy difference between the rhyme/high condition (requiring a “yes” response) and the unrelated condition (requiring a “no” response) of lower than 40% (to ensure that there was no apparent response bias during the tasks). The average reaction time (RT) for each condition was based on correct trials only and was calculated from the onset of the second word for the three experimental word conditions, and the onset of the trial for the perceptual control condition. Reaction times, which were less than or greater than 3 standard deviations from the mean of all correct trials within a run, or were less than 250 ms, were excluded.

### Data Acquisition

Participants lay in the scanner with a response button box placed in their right hand. To keep participants focused on the task, visual stimuli were projected onto a screen, viewed via a mirror attached to the head coil. Participants wore earphones to hear the auditory stimuli, and two pads placed in between the earphones and the head coil were used to reduce movement and attenuate scanner noise.

Images were acquired using a 3.0 T Skyra Siemens scanner with a 64-channel head coil. The blood oxygen level dependent (BOLD) signal was measured using a susceptibility weighted single-shot echo planar imaging (EPI) method. Functional images were acquired with multiband EPI. The following parameters were used: TR = 1,250 ms, TE = 30 ms, flip angle = 80°, matrix size = 128 × 128, FOV = 256 mm^2^, slice thickness = 2 mm without gaps, number of slices = 56, multiband acceleration factor = 4, voxel size = 2 × 2 × 2 mm. A high resolution T1-weighted MPRAGE scan was acquired with the following scan parameters: TR = 1,900 ms, TE = 2.34 ms, matrix size = 256 × 256, FOV = 256 mm^2^, slice thickness = 1 mm, number of slices = 192.

### Data Analysis

Statistical Parametric Mapping 12 (SPM12; Ashburner et al., 2021) was used to analyze the MRI data. First, all functional images were realigned to their mean functional image across runs. The anatomical image was segmented and warped to a pediatric tissue probability map template to get the transformation field. An anatomical brain mask was created by combining the segmented products (i.e., grey, white, and cerebrospinal fluid) and then applied to its original anatomical image to produce a skull-stripped anatomical image. All functional images, including the mean functional image, were then co-registered to the skull-stripped anatomical image. All functional images were then normalized to a pediatric template by applying the transformation field to them and re-sampled with a voxel size of 2 × 2 × 2 mm. The pediatric tissue probability map template was created using CerebroMatic (Wilke et al., 2017), a tool that makes SPM12 compatible pediatric templates with user-defined age, sex, and magnetic field parameters. The unified segmentation parameters estimated from 1,919 participants (Wilke et al., 2017; parameters downloaded from Universitätsklinikum Tübingen, 2022) were used. We defined our parameters as a magnetic field strength of 3.0 T, age range from 9 to 11 years old with one-month intervals, and sex as two females and two males at each age interval to obtain our age-appropriate pediatric template. After normalization, smoothing was applied to all the functional images with a 6 mm isotropic Gaussian kernel.

To reduce movement effects on the brain signal, Art-Repair (Mazaika et al., 2009) was used to identify outlier volumes, which were defined as those with volume-to-volume head movement exceeding 1.5 mm in any direction, head movements greater than 5 mm in any direction from the mean functional image across runs, or deviations of more than 4% from the mean global signal intensity. These outlier volumes were then repaired using interpolation based on the nearest non-outlier volumes. Participants included in our study had no more than 10% of the volumes and no more than six consecutive volumes repaired within each run. The movement criteria were based on those used in previous studies using the same experimental design (e.g., Wang, Yamasaki, et al., 2021; Weiss et al., 2018). Generally speaking, these criteria are more stringent than those used by other research with younger cohorts (e.g., 5- to 7-year-old children by Mathur et al., 2020; 8-year-old children by Girard et al., 2022) and less stringent than those used by other research with older children (e.g., 6- to 19-year-old children by Baker et al., 2020). Six motion parameters estimated during realignment were entered during first level modeling as regressors of no interest, and the repaired volumes were deweighted.

Statistical analyses at the first level were calculated using an event-related design. A high pass filter with a cutoff of 128 s and an SPM default mask threshold of 0.5 were applied. All experimental trials were included as individual events for analysis and modeled using a canonical hemodynamic response function (HRF). All four conditions in each task run (i.e., onset, rhyme, unrelated and perceptual in the sound judgment task, and low, high, unrelated and perceptual in the meaning judgment task) were taken as regressors of interest and entered into the general linear model. We compared the related conditions (i.e., Onset + Rhyme) with the perceptual condition during the sound judgment task to obtain the brain activation map for phonological processing within each participant. We compared the related conditions (i.e., Low + High) with the perceptual condition during the meaning judgment task to obtain the brain activation map for semantic processing within each participant. To examine neural specialization within each participant, we compared brain activation between the two tasks (the sound task (Related > Perceptual) > the meaning task (Related > Perceptual), or the meaning task (Related > Perceptual) > the sound task (Related > Perceptual)]. To examine the parametric modulation effect within each participant, we contrasted the two Related conditions within each task (Onset > Rhyme in the sound task, and Low > High in the meaning task).

In the second-level analyses, task comparison contrast maps from each individual (i.e., the sound task > the meaning task, or the meaning task > the sound task) were entered into a one-sample *t* test to generate a brain specialization map at the group level for either phonological or semantic processing. Contrast maps for the parametric modulations from each individual (i.e., Onset > Rhyme or Low > High) were also entered into a one sample *t* test to generate a parametric modulation map at the group level for either phonological or semantic processing. We used the SPM12 small volume FWE correction to determine the significance of a cluster within our functional language mask. The functional language mask reflects the union of activation for the Related > Perceptual in the sound and meaning judgment tasks within a literature-based anatomical mask. The literature-based anatomical mask included the left IFG, STG, MTG, SMG, AG, IPL, and FG (Binder et al., 2009; Friederici & Gierhan, 2013; Hickok & Poeppel, 2007) and is consistent with the mask used by Weiss et al. (2018) and Wang, Yamasaki, et al. (2021) with younger children. Results at the whole brain level were also calculated using the SPM12 FWE correction.

In addition, exploratory analyses were performed to detect whether the strength of phonological and semantic specialization was associated with children’s language skill. Specifically, we carried out two voxel-wise regression analyses to examine the correlation between brain activation for the contrast Sound (Related > Perceptual) > Meaning (Related > Perceptual) and raw scores on the Phoneme Isolation subtest of the CTOPP-2 (Wagner et al., 2013) and the correlation between brain activation for the contrast Meaning (Related > Perceptual) > Sound (Related > Perceptual) and raw scores on the Word Classes subtest of the CELF-5 (Wiig et al., 2013).

## RESULTS

Table 2 shows the mean, standard deviation, and range of the accuracies and RTs for each condition during the sound and the meaning judgment fMRI tasks. All children included in the study had above chance level (i.e., 50%) overall performance for each task (mean_sound_ = 88.7%, *t*(60) = 45.83, *p* < 0.001; mean_meaning_ = 90.8%, *t*(60) = 48.94, *p* < 0.001). In addition, the hard condition showed significantly lower accuracy and longer RTs than the easy condition in both the sound (*t*(60)_ACC_ = 9.587, *p* < 0.001, *t*(60)_RT_ = −3.086, *p* = 0.003) and the meaning (*t*(60)_ACC_ = 3.448, *p* = 0.001, *t*(60)_RT_ = −6.087, *p* < 0.001) judgment tasks, supporting the validity of the parametric manipulation in the current study.

**Table 2.** Behavioral performance during the sound and the meaning judgment tasks

Tasks	Conditions	Accuracy (%)		Reaction time (ms)	
		Mean (*SD*)	Range	Mean (*SD*)	Range
Sound task	Onset	75.8 (15.1)	29.1–92.4	1,240 (179)	924–1,651
	Rhyme	91.7 (8.5)	66.7–100	1,191 (160)	933–1,565
	Unrelated	89.3 (8.6)	62.5–100	1,296 (190)	1,002–1,749
	Perceptual	97.8 (3.1)	87.5–100	1,225 (451)	516–2,267
Meaning task	Low	86.7 (11.7)	54.2–100	1,244 (153)	832–1,663
	High	90.7 (9.6)	58.3–100	1,158 (164)	799–1,559
	Unrelated	88.2 (10.3)	62.5–100	1,325 (162)	934–1,736
	Perceptual	97.4 (4.0)	83.3–100	1,263 (487)	566–2,478

**Figure 1.**
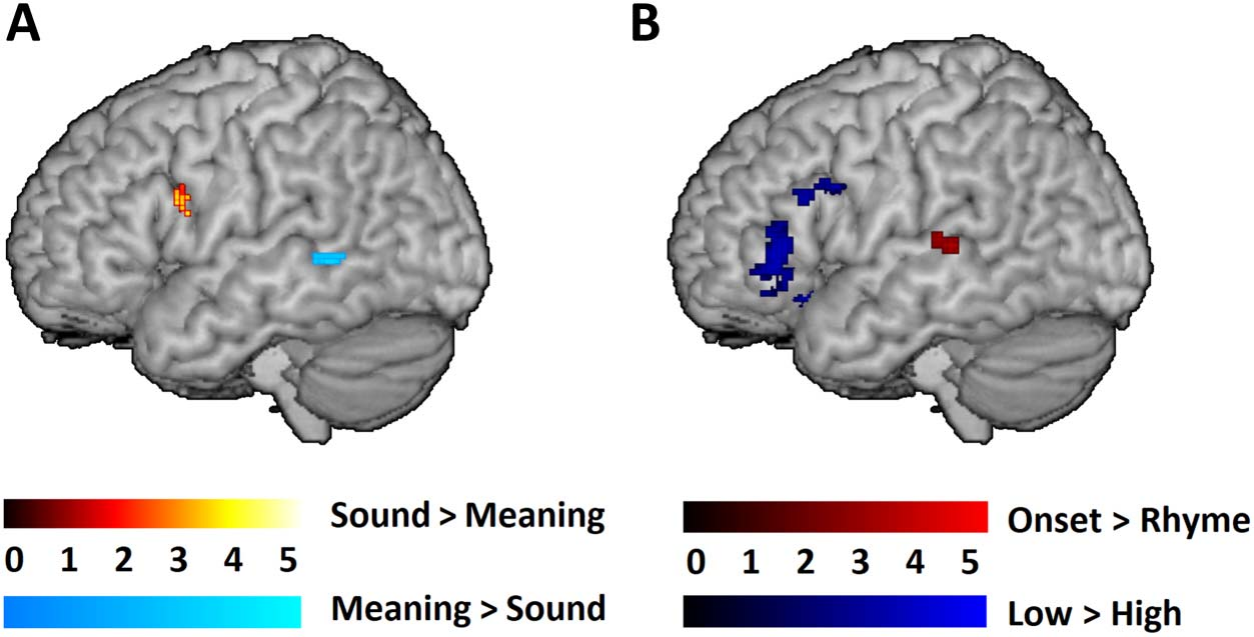
The univariate voxel-wise results within the combined functional and literature-based anatomical mask. (A) Task comparisons: Sound (Related > Perceptual) > Meaning (Related > Perceptual) in hot colors; Meaning (Related > Perceptual) > Sound (Related > Perceptual) in cold colors. (B) Parametric manipulations: Onset > Rhyme within the sound task in red; Low > High within the meaning task in blue. All clusters were significant at a voxel-wise *p* < 0.001 uncorrected, and a cluster-wise *p* < 0.05 family-wise-error corrected, using the SPM12 small volume correction.

The preregistered (https://osf.io/5p3es/) univariate voxel-wise results within the language mask for the direct task comparisons and parametric manipulations are shown in Table 3 and Figure 1. The direct comparisons between the sound and meaning judgment tasks (see Figure 1A) revealed significantly greater activation for the sound judgment task in the left opercular part of the IFG and significantly greater activation for the meaning judgment task in the left MTG. (Brain specialization maps after controlling covariates of no interest including task accuracy, nonverbal IQ, and core language skill are displayed in Table S1 and Figure S1 in the Supporting Information. The main findings remained the same except that the opercular part of the left IFG was no longer significant for the contrast of Low > High in the meaning task.) In terms of the parametric manipulations, the comparison between the onset and rhyme conditions within the sound judgment task showed significant clusters in the left STG/STS (superior temporal sulcus; see Figure 1B). The comparison between the low and high association conditions within the meaning judgment task showed significant clusters in both the triangular/orbitalis and opercular part of the IFG (see Figure 1B). As is shown in Figure 1, the significant clusters found in the parametric manipulations did not overlap with the significant clusters found in the direct task comparisons. Finally, the regression analyses for the preregistered exploratory analyses did not reveal any significant clusters between specialization-related brain activation and phonological awareness or semantic association skill as measured by the standardized tests.

**Table 3.** Voxel-wise analysis significant results within the combined functional and literature-based anatomical mask

Brain regions	Brodmann area	Peak coordinate (MNI)	Number of voxels	*T* value
Sound task (Related > Perceptual) > Meaning task (Related > Perceptual)				
Opercular left IFG	44	−42 2 24	103	5.06
Meaning task (Related > Perceptual) > Sound task (Related > Perceptual)				
Left MTG	21	−58 −46 0	37	4.28
Onset > Rhyme in the sound task				
Left STG/STS	22	−66 −32 6	39	3.69
Low > High in the meaning task				
Triangular/orbitalis left IFG	45/47	−54 32 2	288	4.40
Opercular left IFG	44	−50 12 28	71	4.09

*Note*. IFG = inferior frontal gyrus, MTG = middle temporal gyrus, STG = superior temporal gyrus, STS = superior temporal sulcus, MNI = Montreal Neurological Institute.

**Figure 2.**
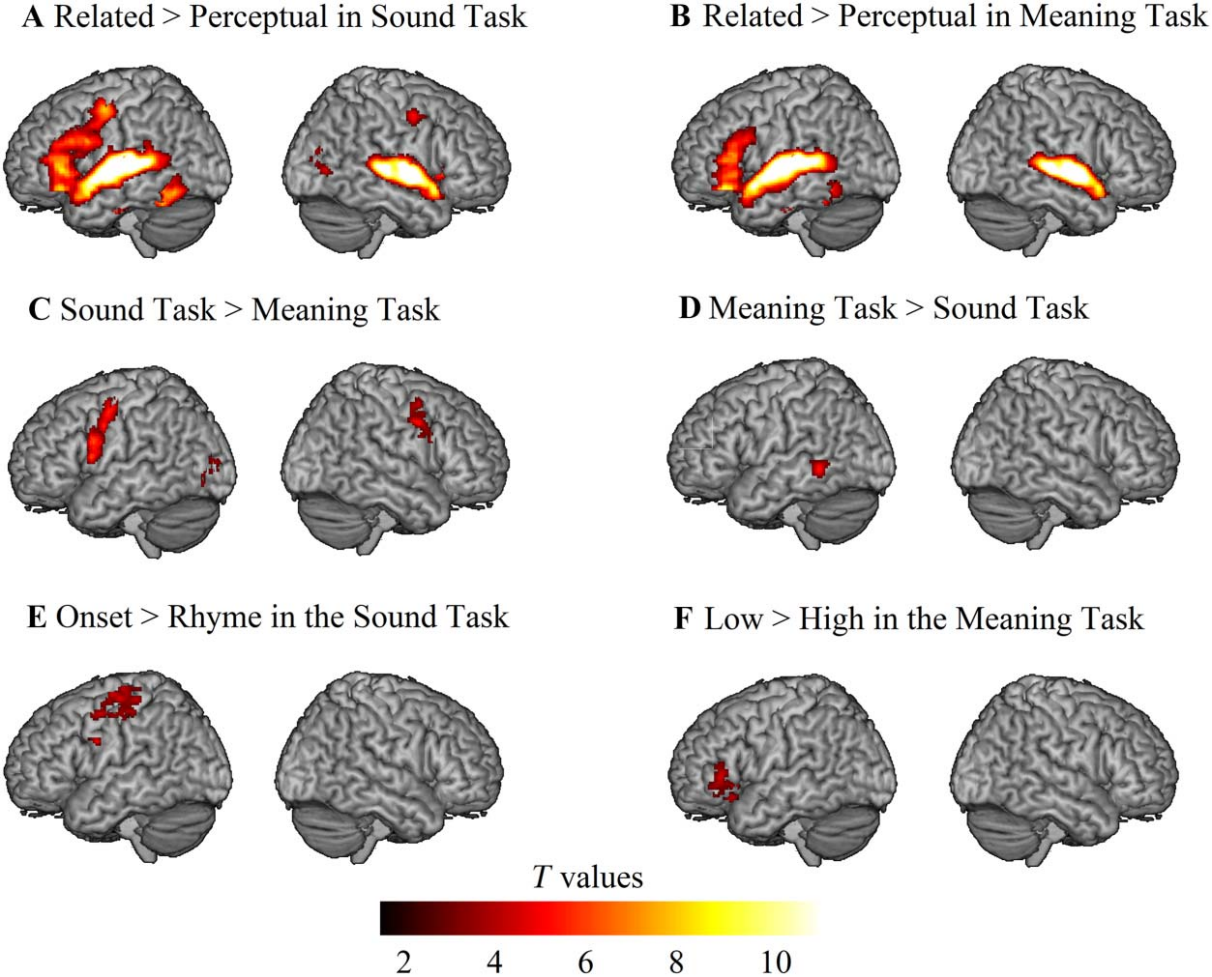
The univariate voxel-wise results at the whole-brain level. (A) Related > Perceptual in the Sound Task; (B) Related > Perceptual in the Meaning Task; (C) Sound Task (Related > Perceptual) > Meaning Task (Related > Perceptual); (D) Meaning Task (Related > Perceptual) > Sound Task (Related > Perceptual); (E) Parametric manipulation in the Sound Task (Onset > Rhyme); (F) Parametric manipulation in the Meaning Task (Low > High). All clusters were significant at a voxel-wise *p* < 0.001 uncorrected, and cluster-wise *p* < 0.05 family-wise-error corrected in SPM12 at the whole brain level.

The non-preregistered univariate voxel-wise results at the whole brain level for each task, the direct task comparisons, and the parametric manipulations are displayed in Table 4 and Figure 2. We focused our summary of results on the regions of interest specified in the literature-based anatomical mask, including their homologues in the right hemisphere. We found that the left IFG, bilateral STG/MTG, and bilateral FG were commonly activated across the sound (Figure 2A) and meaning (Figure 2B) judgment tasks. Direct task comparisons did not reveal clusters in the literature-based anatomical areas that showed greater activation for the sound task (Figure 2C), whereas the meaning task induced greater activation in the left MTG (Figure 2D). As for the parametric manipulation effects, no clusters in the literature-based anatomical areas showed greater activation for the onset than the rhyme condition within the sound judgment task (Figure 2E), whereas the orbitalis/triangular part of the left IFG exhibited greater activation for the low than the high association condition within the meaning judgment task (Figure 2F). In terms of the regression analysis, we did not find any significant clusters showing a correlation between brain activation for specialization and language skills as measured by standardized testing at the whole brain level.

**Table 4.** Voxel-wise analysis significant results at the whole brain level

Brain regions	Brodmann area	Peak coordinate (MNI)	Number of voxels	*T* value
Sound task (Related > Perceptual)				
Left STG	22	−62 8 0	7725	17.04
Right STG	22	62 −6 −4	2691	14.19
Left fusiform	37	−42 −44 −16	1648	9.16
Right insula	13	32 20 2	255	6.79
Left putamen	–	−16 8 6	1654	6.73
Left thalamus	–	−10 −16 6	110	6.51
Left supplementary motor area	6	−6 6 60	431	5.64
Right lingual gyrus	19	16 −48 4	136	5.15
Left calcarine	17	−16 −50 8	131	5.06
Right precentral gyrus	6	54 −4 46	118	5.03
Right MTG	21	56 −74 2	132	4.77
Meaning task (Related > Perceptual)				
Right MTG/STG	21/22	66 −4 −2	2659	14.79
Left STG/MTG/IFG	22/21	−64 −10 2	5369	14.08
Left inferior temporal gyrus	20	−38 −14 −28	1338	8.07
Right para-hippocampus	–	22 −6 −22	172	5.16
Sound task (Related > Perceptual) > Meaning task (Related > Perceptual)				
Left precentral gyrus	6	−58 6 24	953	5.86
Right precentral gyrus	6	52 2 36	307	4.57
Left middle occipital lobe	19	−42 −90 10	108	4.03
Meaning task (Related > Perceptual) > Sound task (Related > Perceptual)				
Left MTG	21	−62 −46 −4	144	5.20
Onset > Rhyme in the sound task				
Left postcentral gyrus	4	−34 −10 30	115	4.99
Left insula	13	−22 26 16	141	4.74
Left caudate	–	−16 −2 26	142	4.74
Left precentral gyrus	3	−40 −18 62	502	4.10
Low > High in the meaning task				
Left orbitalis/triangular IFG	47/45	−32 22 −4	389	4.74
Left supplementary motor area	8	−6 20 50	131	4.13

## DISCUSSION

This study examined phonological and semantic specialization in 9- to 10-year-old children using the same experimental design and analytical approach as used in previous studies with younger children aged 5 to 6 years old (Weiss et al., 2018) and 7 to 8 years old (Wang, Yamasaki, et al., 2021). Similar to the findings with 7- to 8-year-old children, the current study showed phonological and semantic specialization in both the frontal and temporal lobes. Specifically, the left dIFG showed greater activation for the sound than the meaning judgment task, whereas the left MTG showed greater activation for the meaning than the sound judgment task. In terms of the parametric manipulations, we found that the left STG showed greater activation for the onset than the rhyme condition during the sound judgment task, whereas both the left vIFG and the opercular part of the left IFG showed greater activation for the low than high association condition during the meaning judgment task. Overall, this pattern of results suggests that, despite a few differences, phonological and semantic specialization in 9- to 10-year-old children remains similar to that observed in 7- to 8-year-old children.

The current study found that the left dIFG showed greater activation for the sound than the meaning task in 9- to 10-year-old children, showing support for phonological specialization in the frontal lobe. This finding is consistent with a previous study on 7- to 8-year-old children (Wang, Yamasaki, et al., 2021), which also showed phonological specialization in the frontal but not the temporal lobes. Phonological specialization in the temporal lobe was only observed in a study with even younger children aged 5 to 6 years old (Weiss et al., 2018), in which the left STG showed greater activation for the sound than the meaning judgment task. It has been hypothesized that the left STG is associated with the quality of phonological representation, whereas the left dIFG is associated with phonological access and manipulation (e.g., Boets et al., 2013; Myers et al., 2009). Together with the previous studies on younger children (Wang, Yamasaki, et al., 2021; Weiss et al., 2018), the current study suggests a developmental trajectory of phonological specialization from the temporal to the frontal lobe. Previous research has also shown that younger children vary in the representational quality of phonological forms in STG as a function of skill (e.g., Wang et al., 2020), whereas older children vary in the efficiency of phonological access in IFG as a function of skill (Wang, Pines, et al., 2021). It is likely that although both representation and access are needed to perform phonological awareness tasks, young children rely more on the quality of phonological representation in the left STG, whereas children older than 7 to 8 years old consistently rely more on the efficiency of phonological access and manipulation in the left dIFG.

However, unlike the left dIFG observed in the previous study on 7- to 8-year-old children, which was localized to the triangular part of the left IFG (Wang, Yamasaki, et al., 2021), the left dIFG observed in the current study on 9- to 10-year-old children was in the opercular part of the left IFG. Previous research suggests that the opercular part of the left IFG functions as a domain-general control region and is engaged in difficult tasks (e.g., Hodgson et al., 2021). Consistent with this interpretation, the overall accuracy for the sound task was significantly lower than that for the meaning task (85.6% for the sound task, 88.5% for the meaning task, *t* = −2.905, *p* = 0.005), suggesting that the sound task was more difficult than the meaning task. However, when task accuracy was controlled for, the opercular part of the left IFG was still significantly more active for the sound than the meaning task, suggesting its role specifically in phonologically related control processes. In line with this argument, previous research has shown that the opercular part of the left IFG is a core region for phonological processing, whereas the triangular part of the left IFG is involved in semantic processing in adults (e.g., Costafreda et al., 2006; Katzev et al., 2013; Poldrack et al., 1999). Thus, the progression of phonological specialization from the triangular to opercular part of the left IFG likely suggests that phonological access and manipulation in 9- to 10-year-old children, as compared to 7- to 8-year-old children, is more adult-like and, potentially, more effective. In support of this argument, we observed that the mean accuracies for the onset and rhyme conditions (i.e., onset: 76%, rhyme: 92%) in the current study were higher than those found for 7- to 8-year-old children (i.e., onset: 70%, rhyme: 88%; Wang, Yamasaki, et al., 2021). However, because the current study is cross-sectional, future studies with longitudinal designs are needed to examine the developmental trajectory that is suggested by the combination of results from the current study and previous work.

As for semantic specialization, we found that the left MTG showed greater activation for the meaning than the sound judgment task. Previous research has already shown that the left MTG is a semantic processing region and is reliably engaged in the analysis of semantic relatedness (e.g., Binder et al., 2009; Enge et al., 2021). Skeide et al. (2014) also found that the temporal lobe shows gradual specialization for semantic processing and away from syntactic processing from 7 to 8 years old. Thus, the finding of semantic specialization in the left MTG in the current study is not surprising. This finding is also consistent with the study by Liu et al. (2012) with 11- to 13-year-old children using visual tasks and previous studies using the same experimental design and analytical approach with 7- to 8-year-old children (Wang, Yamasaki, et al., 2021) and 5- to 6-year-old children (Weiss et al., 2018), in which children also showed greater activation for the meaning than the sound judgment task in the left MTG, at a similar location. However, unlike the previous study on 7- to 8-year-old children (Wang, Yamasaki, et al., 2021), the current study did not find that the left vIFG showed greater activation for the meaning than the sound judgment task in 9- to 10-year-old children. The lack of a significant finding in the left vIFG may be a power issue. When the statistical threshold was lowered from voxel-wise *p* < 0.001 to voxel-wise *p* < 0.005, we found a cluster showing greater activation for the meaning than the sound judgment task in the orbitalis/triangular part of the left IFG (peak MNI −46 34 −14, *k* = 17), a similar location to that found previously with 7- to 8-year-old children. Because we did not conduct an a priori power analysis it is difficult to determine if the lack of the finding is due to a lack of power or reflects no effect, and more research is needed to examine the replicability of this weak finding. In contrast to the observed frontal specialization for phonological processing, the lack of observed semantic specialization in the frontal lobe in 9- to 10-year-old children may have also been driven by the fact that the meaning judgment task was easier than the sound judgment task, as the frontal lobe is known to be engaged particularly when tasks are more difficult or demanding (e.g., Burton et al., 2000; Chiou et al., 2018; Katzev et al., 2013; Okada et al., 2018; Xie & Myers, 2018). Overall, the similarity in the pattern of findings for semantic specialization across multiple studies (e.g., Liu et al., 2012; Wang, Yamasaki, et al., 2021; Weiss et al., 2018) likely suggests that the semantic system remains stable over middle childhood. This argument is supported by a previous meta-analysis on the semantic system in developing children ages 4 to 15 years old (Enge et al., 2021), in which they found little evidence for age-related changes across childhood and high overlap with the adult semantic system.

In terms of the parametric manipulations, we found that the left STG showed greater activation for the onset than the rhyme condition during the sound judgment task. This finding is consistent with previous studies with 7- to 8-year-old and 5- to 6-year-old children (Wang, Yamasaki, et al., 2021; Weiss et al., 2018) showing that the left STG was more engaged for the onset than the rhyme condition during the sound judgment task. The left STG has been consistently shown to be associated with phonological representation (Boets et al., 2013; Mesgarani et al., 2014) and was found engaged more for onset than rhyme processing in children with higher phonological skill (e.g., Wang, Joanisse, & Booth, 2021). Thus, it is unsurprising that the onset condition, which requires more precise phonological representations, engaged the left STG more than the rhyme condition during the sound judgment task. However, somewhat surprisingly, we did not observe any significant clusters in the left IFG in 9- to 10-year-old children for the phonological parametric manipulation. This is in contrast to the previously reported finding that in 7- to 8-year-old children the left opercular part of the left IFG was more active for the onset than the rhyme condition. The engagement of the opercular part of the left IFG found in 7- to 8-year-old children was hypothesized to be reflective of a general cognitive control process because both the sound and the meaning tasks elicited parametric effects in this area and direct task comparisons revealed no activation difference. The lack of a significant parametric effect in the opercular part of the left IFG in the current study might suggest that 9- to 10-year-old children rely on other mechanisms to deal with the more difficult onset judgment. At the whole brain level, the left precentral gyrus was consistently activated during the sound judgment task and was more activated for the onset than the rhyme condition (although at a more dorsal location). This area is close to the Exner’s area, a region associated with handwriting, bridging orthographic and motion systems (Roux et al., 2009). Although children’s phonemic awareness could appear as early as 4 to 5 years old (Anthony & Francis, 2005), learning to read connects written and spoken languages and thus sculpts the nature of phonological processing in the brain (e.g., Wang et al., 2020; Wang, Pines, et al., 2021). Therefore, 9- to 10-year-old children in the current study may have employed a strategy of spelling out the spoken words to help perform the more fine-grained phonological awareness task (i.e., the onset judgment). However, given that we did not have a spelling measurement to confirm the function of this area, more studies are needed to examine this speculation.

For the semantic parametric manipulation analysis, we found that both the left vIFG and the left opercular part of the left IFG showed greater activation for the low compared to the high association condition during the meaning judgment task. Previous literature on adults and children (e.g., Poldrack et al., 1999; Wang, Yamasaki, et al., 2021) has already shown that the left vIFG is a region specialized for semantic processing, and that, in adults, it is more engaged when semantic associations are atypical or more demanding (e.g., Chiou et al., 2018; Katzev et al., 2013). In the current study, children performed significantly worse in the low than the high association condition (*t*(60) = 3.45, *p* = 0.001), suggesting that the low association condition was more difficult. This difference in difficulty between conditions may be driving the engagement of the opercular part of the left IFG, as it is a general cognitive control region often recruited to promote a non-automatic but appropriate response (e.g., Novick et al., 2010). In comparison with the previous finding that 7- to 8-year-old children only recruited the opercular part of the left IFG for the low association condition (Wang, Yamasaki, et al., 2021), the additional finding of a parametric effect in a semantically specialized region (i.e., the left vIFG) in the current study suggests that 9- to 10-year-old children start to engage more task-specific regions to solve more difficult semantic problems.

It is interesting to note that when looking at the parametric effects for the sound and meaning judgment tasks together, the patterns of activation do not overlap. This may suggest that 9- to 10-year-old children rely more on task-specific regions to deal with more difficult language tasks, which is different from the previous findings with 7- to 8-year-old children (Wang, Yamasaki, et al., 2021), where the parametric effects were largely overlapping in the opercular part of the left IFG. To examine if the parametric effects observed in each task were task-specific or domain-general, additional analyses comparing the parametric effects across tasks were conducted. In 7- to 8-year-old children, Wang, Yamasaki, et al. (2021) showed no task differences, suggesting that those children tended to use the same general control mechanisms to tackle more difficult language problems. However, using the same analytical approach, it was found, in the current study, that the left STG (peak MNI −60 −18 12, *k* = 132) was significantly more activated for the sound (Onset > Rhyme) than the meaning (Low > High) parametric manipulation. In addition, a small nonsignificant cluster in the left vIFG (peak MNI −58 30 6, *k* = 2) was found to be activated more for the meaning (Low > High) than the sound (Onset > Rhyme) parametric manipulation. When the voxel-wise threshold was lowered from *p* < 0.001 to *p* < 0.005, we found a relatively big cluster at the same location (peak MNI −58 30 6, *k* = 37) in the left vIFG. The weaker parametric effect in the meaning judgment task as compared to the sound judgment task parallels the accuracy difference between the hard and easy conditions in the meaning judgment task (i.e., 4%, Low: 86.7%, High: 90.7%) versus that of the sound judgment task (i.e., 15.9%, Onset: 75.8%, Rhyme: 91.7%). Overall, these additional analyses help to confirm that unlike 7- to 8-year-old children who tend to rely on a domain-general control system, 9- to 10-year-old children use more language-specific regions to deal with difficult language tasks.

The implication of a developmental progression from reliance on domain-general to domain-specific processing in the current study is consistent with a domain-relative framework for cognitive development (e.g., Karmiloff-Smith, 2015; Sloutsky, 2010), which argues that the infant brain comes equipped with biases that are relevant to, but not initially specific to, processing different kinds of input. Therefore, domain-general learning mechanisms serve as a beginning state and domain-specific mechanisms gradually appear via neural competition and stimulation from the environment. Although this theory has not yet been supported by language specialization studies, this argument has consistently been supported by previous studies on word learning during infancy using computational and behavioral approaches. For example, Mayor and Plunkett (2010) developed a neurocomputational model and showed that domain-specific word learning constraints can emerge out of domain-general, associative learning principles when confronted with a structured environment. Consistent with this argument, Namy (2012) reviewed previous studies and found that word learning started out general and became largely a domain-specific ability over the course of the second year. Similarly, Samuelson and McMurray (2017) argued that domain-general processes played an important role in initial word learning. Although word learning likely draws on different processes than those employed in the semantic association and phonological awareness tasks in the current study, our findings suggest that the underlying neural development during language processing from domain-general to domain-specific is similar. Karmiloff-Smith (1998) argues that understanding development itself is the key to understanding the complexity of developmental language disorders, because deviance in this developmental trajectory is likely to result in a cascade of dynamic subtle deficits across domains rather than a single, static domain-specific one. Our finding of a shift from relying on domain-general to domain-specific mechanisms in 9- to 10-year-old children during more difficult language processing provides critical developmental information. That is, typical children continue to develop domain-specificity during word processing until 9 to 10 years old, before which domain-general ability might play a larger role in word processing, especially during more difficult language tasks.

As for the proposed exploratory analyses, we did not find any significant clusters showing a correlation between brain specialization and language skills, which is consistent with previous finding with 7- to 8-year-old children (Wang, Yamasaki, et al., 2021). According to the memory, unification, and control model by Hagoort (2016), regions in the temporal cortex subserve knowledge representations that have been laid down in memory during acquisition. In contrast, frontal regions, which are structurally and functionally connected to temporal regions, support memory retrieval, decomposition, and unification operations. The lack of a brain-behavioral correlation may be because specialization in the frontal lobe provided children beyond 7 to 8 years old with more flexibility in their division of labor in terms of engaging the representation and control systems during specific language tasks. According to the distributions-to-associations model by Shrager and Siegler (1998), children can either retrieve an answer using their stored knowledge representation or adaptively utilize back-up strategies when facing a problem (Siegler, 1988). Unlike 5- to 6-year-old children, who rely primarily on knowledge representation stored in the temporal lobe (Weiss et al., 2018), children older than 7 to 8 years old might have access to more back-up strategies by engaging their frontal lobes. The larger individual differences in the choice of strategies could lead to more difficulty finding brain-behavior correlations in older children than in younger children. In addition, behavioral performance on standardized tests is a byproduct of multiple cognitive processes, including attention and working memory. However, brain specialization, as operationalized in the current study as a double dissociation between tasks, reflects language-specific processes. Direct task comparisons of brain activity between two similar tasks within a language mask allowed us to cancel out irrelevant processes that were shared by the two tasks (e.g., attention, working memory) and extract brain activity that is specific to language processing. This mismatch of the underlying processes measured by behavioral performance and brain activity reduces the likelihood of finding brain-behavior correlations. Although we do not know how skill is related to language specialization observed in 9- to 10-year-old children, as compared to younger children (Wang, Yamasaki, et al., 2021; Weiss et al., 2018), older children clearly showed increased specialization, which likely means that children’s brains become increasingly modularized into regions with unique computational principles. The interactive specialization theory (Johnson, 2011) hypothesizes that brain interaction drives specialization. Thus, we think that this increased specialization should be related to interactions across brain regions, but this needs to be systematically investigated with future studies.

In summary, the current study showed phonological and semantic specialization in both the temporal and frontal lobe in 9- to 10-year-old children, similar to that observed in 7- to 8-year-old children. However, different from 7- to 8-year-old children who seem to rely on a general cognitive control region, 9- to 10-year-old children appear to rely on language-specific regions to deal with more difficult language tasks. Overall, this study suggests that at 9 to 10 years old, children’s phonological and semantic specialization in the brain continues to become more adult-like.

## FUNDING INFORMATION

James R. Booth, National Institutes of Health (https://dx.doi.org/10.13039/100000002), Award ID: DC013274.

## AUTHOR CONTRIBUTIONS

**Jin Wang**: Conceptualization; Formal analysis; Writing – original draft; Writing – review & editing. **Brianna L. Yamasaki**: Conceptualization; Writing – review & editing. **James R. Booth**: Conceptualization; Funding acquisition; Writing – review & editing.

## DATA AVAILABILITY STATEMENT

All data were pulled from a published data set on OpenNeuro.org (see detailed description in Wang et al., 2022; https://openneuro.org/datasets/ds003604). The specific subjects and runs used in the current study as well as the code used to analyze the data were shared on GitHub https://github.com/wangjinvandy/PhonSem_Specialization_9_10.

## TECHNICAL TERMS

Specialization: A process where brain regions become more responsive to their preferred task than other tasks.
Semantic: Refers to the meaning structures in language.
Direct task comparison: The contrast of brain activation between similar tasks that differ primarily in a process of interest.
Phonology: Refers to the sound structures in language.
Double dissociation: Occurs when brain region A activates more for Task 1 than Task 2, whereas region B activates more for Task 2 than Task 1.
Domain-general: Brain regions, associated with cognitive-control mechanisms, that are recruited across domains, particularly when tasks are more difficult.
Language-specific: Brain regions that are recruited only during language tasks, and not others.
