# Supplementary material for: Phonological and Semantic Specialization in 9- to 10-Year-Old Children During Auditory Word Processing
Source: Neurobiol Lang (Camb). 2023 Apr 11;4(2):297–317. doi: 10.1162/nol_a_00099 (PMC10205156; doi:10.1162/nol_a_00099)
Supplement: Supplementary file 1 [file nol-4-2-297-s001.docx]

Supplementary Materials

Table S1. Significant results for the voxel-wise analysis within the combined functional and literature-based anatomical mask after controlling covariates of no interest including task accuracy, non-verbal IQ and core language skill.

| Brain regions | Brodmann Area | Peak Coordinate (MNI) | Number of voxels | T value |
| --- | --- | --- | --- | --- |
| Sound Task (Related > Perceptual) > Meaning Task (Related > Perceptual) | | | | |
| Opercular left IFG | 44 | -42 2 24 | 100 | 5.44 |
| Meaning Task (Related > Perceptual) > Sound Task (Related > Perceptual) | | | | |
| Left MTG | 21 | -58 -46 0 | 42 | 4.28 |
| Onset > Rhyme in the Sound Task | | | | |
| Left STG/STS | 22 | -66 -32 6 | 66 | 4.54 |
| Low > High in the Meaning Task | | | | |
| Triangular left IFG | 45/47 | -34 24 -6 | 274 | 4.10 |


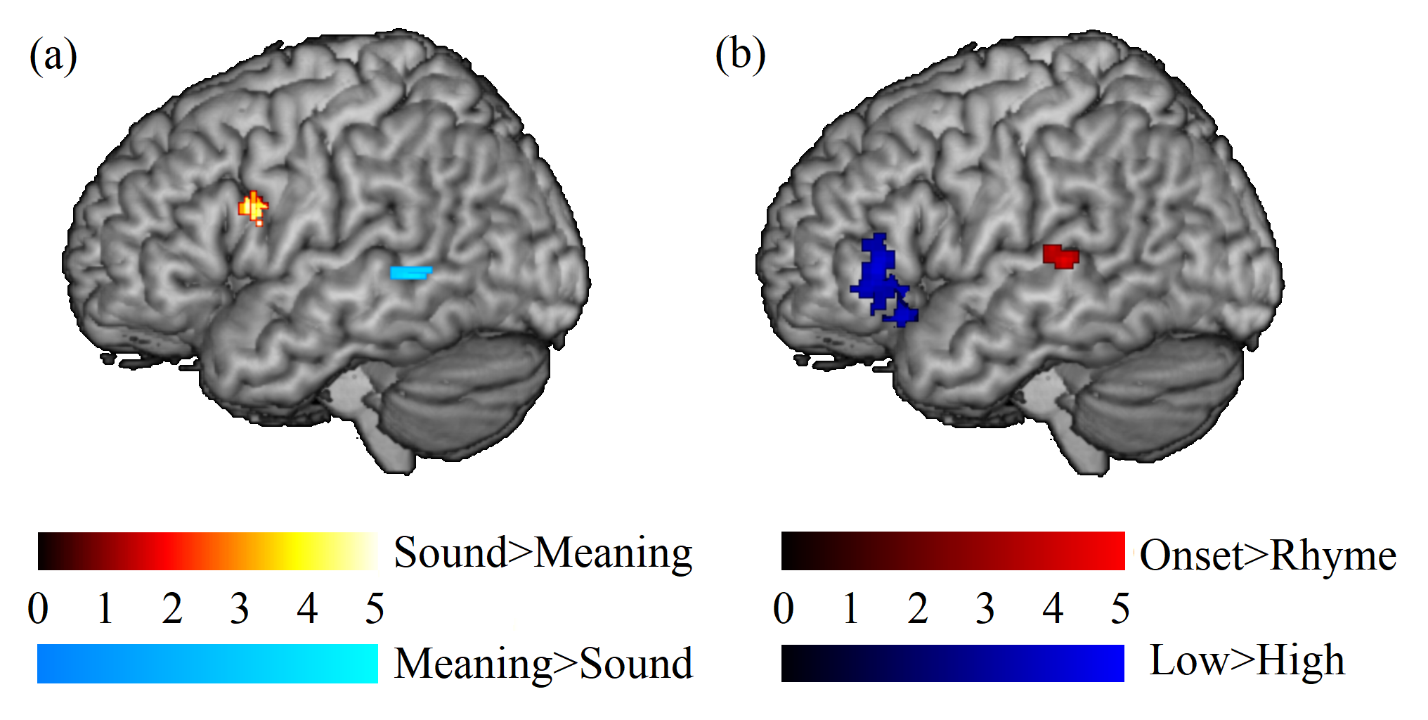


Figure S1. Voxel-wise analysis significant results within the combined functional and literature-based anatomical mask after controlling covariates of no interest such as task accuracy, non-verbal IQ and core language skill. (a) Task comparisons: Sound Task (Related > Perceptual) > Meaning Task (Related > Perceptual) in hot colors; Meaning Task (Related > Perceptual) > Sound Task (Related > Perceptual) in cold colors. (b) Parametric manipulations: Onset > Rhyme within the Sound Task in red; Low > High within the Meaning Task in blue. All clusters were significant at a voxel-wise p < 0.001 uncorrected, and a cluster-wise p < 0.05 family-wise-error corrected, in SPM12 small volume correction.

Stimuli

| Sound Task | Onset | | Rhyme | | Unrelated | |
| --- | --- | --- | --- | --- | --- | --- |
| run | word_1 | word_2 | word_1 | word_2 | word_1 | word_2 |
| 1 | coat | cup | wide | ride | zip | cone |
| 1 | nut | nail | gown | town | bug | hip |
| 1 | pail | poke | bed | red | boat | hum |
| 1 | gap | gift | name | blame | feed | belt |
| 1 | rock | roast | dash | smash | land | face |
| 1 | sell | sing | kid | slid | rent | soup |
| 1 | yeast | yell | brave | save | build | saint |
| 1 | dump | deck | plug | mug | pan | truck |
| 1 | bond | bake | stall | call | fresh | need |
| 1 | haunt | help | cliff | sniff | stove | map |
| 1 | calm | coast | brain | plain | snail | crook |
| 1 | lend | lump | trip | slip | cloth | brown |
| 2 | rug | rap | rake | cake | kiss | log |
| 2 | sock | safe | bad | mad | fall | must |
| 2 | mud | meal | pin | win | yawn | silk |
| 2 | goal | gust | dead | bread | desk | feel |
| 2 | tall | toast | mop | stop | gasp | fund |
| 2 | fence | fat | sack | black | bank | halt |
| 2 | ramp | read | prize | size | path | food |
| 2 | paint | pick | stack | pack | hate | rod |
| 2 | mend | mask | speed | weed | dive | sleep |
| 2 | bend | bulb | creep | sweep | bat | slime |
| 2 | sand | salt | snap | trap | grip | line |
| 2 | van | vest | brick | stick | flash | tribe |

| Meaning Task | High association | | Low association | | Unrelated | |
| --- | --- | --- | --- | --- | --- | --- |
| run | word_1 | word_2 | word_2 | word_2 | word_1 | word_2 |
| 1 | syrup | pancakes | water | drink | flush | cliff |
| 1 | trash | garbage | salad | lettuce | sailing | nest |
| 1 | above | below | dish | plate | chill | hawk |
| 1 | yell | scream | dig | shovel | adult | pond |
| 1 | mitten | glove | little | big | cobra | slipper |
| 1 | spoon | fork | call | phone | trade | square |
| 1 | touch | feel | picture | painting | blanket | split |
| 1 | table | chair | purse | wallet | flow | teaching |
| 1 | itch | scratch | children | kids | bacon | wipe |
| 1 | tall | short | throw | catch | bone | mountain |
| 1 | sketch | draw | silent | quiet | corn | blame |
| 1 | washer | dryer | sneeze | cough | dessert | bony |
| 2 | summer | winter | lobster | crab | turtle | boom |
| 2 | silver | gold | sunny | cloudy | map | hut |
| 2 | dog | cat | skunk | stink | towel | puppet |
| 2 | bunny | rabbit | human | person | rich | pointed |
| 2 | mom | dad | checkers | chess | trust | dozen |
| 2 | start | finish | finish | end | plug | belly |
| 2 | fingers | hands | maple | syrup | spell | dim |
| 2 | ship | boat | cheese | cracker | hearing | bottle |
| 2 | winner | loser | save | keep | hoof | stitch |
| 2 | awake | asleep | hop | skip | pirate | bandage |
| 2 | question | answer | lip | kiss | jewel | moan |
| 2 | web | spider | hide | seek | system | head |
